# Research progress on immune checkpoint inhibitors in intrahepatic cholangiocarcinoma

**DOI:** 10.1080/07853890.2025.2584667

**Published:** 2025-11-14

**Authors:** Ziwen Yin, Lin Zhang, Jiexu Li, Haiyang Tan, Bingqi Ma

**Affiliations:** a Department of Hepatopancreatobiliary Surgery, Affiliated Hospital of Shandong Second Medical University, Weifang, China; b Department of Pharmacy, Affiliated Hospital of Shandong Second Medical University, Weifang, China

**Keywords:** Intrahepatic cholangiocarcinoma, tumor microenvironment, immune checkpoint inhibitor, immunotherapy

## Abstract

**Background:**

Intrahepatic cholangiocarcinoma (ICC) is a relatively uncommon but lethal neoplasm of the biliary tract. Evolving immunotherapy has important implications for the treatment of ICC. Novel immune checkpoint inhibitors (ICIs) continue to be identified and have demonstrated clinical efficacy in the treatment of ICC, though there are some issues.

**Objective:**

This article profoundly discusses the core issues of immunotherapy for ICC, provides a comprehensive and systematic overview, and propose corresponding countermeasures to improve patient outcomes .

**Methods:**

We analyze the tumor microenvironment to validate the important role of immune checkpoints in tumor immune escape. By incorporating multiple clinical and preclinical trials to summarize the latest advances in ICIs for ICC treatment, we investigate the current challenges and limitations of ICC immunotherapy and explore new directions for its development.

**Results:**

Monotherapy approaches fail to achieve satisfactory outcomes in ICC. Combination therapies, such as the pairing of ICIs with chemotherapy, exhibit synergistic effects, overcome drug resistance and enhance therapeutic efficacy. Furthermore, targeted therapies, such as tyrosine kinase inhibitors and IDH inhibitors, have exhibited antitumor activity in the context of ICC.

**Conclusion:**

In summary, ICIs are assuming an increasingly prominent role in the management of ICC. Efforts should be made to improve treatment methods and apply combination therapy strategies to address drug resistance. The combination of multiple treatment methods and ongoing research are crucial for addressing the treatment of ICC.

## Introduction

Intrahepatic cholangiocarcinoma (ICC) is a highly lethal, aggressive, and heterogeneous malignancy of the biliary ducts [1]. The mortality rate of ICC has remained consistently high, with a five-year overall survival (OS) rate remaining around 9% [2]. Surgical resection remains the only potential method of cure at present [3], but is limited to early patients [4,5]. Due to its nonspecific symptoms such as fatigue, weight loss, abdominal pain, obstructive jaundice, most patients are already in the progressive stage at the time of diagnosis, and are unable to undergo radical surgical resection [6]. Following the ABC-02 trial, the GC (Gemcitabine + Cisplatin) regimen has been swiftly adopted in clinical practice, becoming a first-line treatment option for advanced biliary tract cancer (BTC) [7]. Several alternative regimens have also been validated to enhance outcomes in ICC, including GEMOX (Oxaliplatin + Gemcitabine) and the GCS regimen (Gemcitabine + Cisplatin + S-1) [8,9]. Nevertheless, the benefit is minimal of these regimens. A long-term survival trend analysis of BTC patients receiving chemotherapy revealed that over the past two decades, the utilization of GC regimen as a first-line treatment has increased substantially (by 34.56%), yet median overall survival (mOS) shows no marked change [10]. The overall efficacy of other regimens such as GEMOX and fluoropyrimidine-based regimens also remains limited, with 45.2% of patients experiencing disease progression [10]. While the ABC-06 trial established the mFOLFOX regimen as the standard second-line treatment for advanced BTC (mOS: 6.2 months), the benefit it provided was minimal, with mOS prolonged by merely 0.9 months [11,12]. Tumor progression and disease deterioration can also impact chemotherapy efficacy. A meta-analysis showed that the cachexia index (CXI) positively correlates with patient OS, indirectly supporting this observation [13]. Accumulating research evidence has shown that immune-inflammatory markers like the neutrophil-lymphocyte ratio (NLR) [14], neutrophil-to-eosinophil ratio (NER) [15], and prognostic inflammatory index (PII) [16] can also effectively predict ICC prognosis, thereby providing compelling evidence linking the occurrence and development of ICC to immune-related factors. Concurrently, these factors can function as predictors to guide treatment selection for ICC patients. Conventional treatments have limited efficacy and significant side effects. Therefore, exploring new treatment regimens is crucial for improving the prognosis of patients with advanced ICC.

ICIs, including but not limited to anti-cytotoxic T-lymphocyte-associated protein 4 (CTLA-4), anti-programmed death-1 (PD-1) and anti-programmed death-1 ligand 1 (PD-L1), have demonstrated significant efficacy in the treatment of various cancers. For patients diagnosed with advanced head and neck squamous cell carcinoma, non-small cell lung cancer (NSCLC), melanoma, gastric cancer, and hepatocellular carcinoma (HCC), ICIs have become a component of the standard treatment regimen [17–19]. Moreover, ongoing trials such as TOPAZ-1 and EYNOTE-966 have also revealed their efficacy as new first-line treatment in ICC [20,21]. Therefore, we review and summarize the current research on ICIs in ICC and the evidence supporting its role in combination therapy for ICC, with the aim of optimizing treatment strategies for ICC patients and improving their survival outcomes.

## Tumor microenvironment of ICC

The tumor microenvironment (TME), which plays a pivotal role in the survival, invasion, and metastasis of malignancy, is constituted by tumor cells colonizing normal tissues, stromal cells (e.g. cancer-associated fibroblasts [CAFs]), immune cells (e.g. tumor-associated macrophages [TAMs], regulatory T-lymphocytes [Tregs], natural killer [NK] cells), cytokines, vascular endothelial cells, and the extracellular matrix [22,23]. During the progression of tumors, the formation of an immunosuppressive TME is driven by six core characteristics: compositional heterogeneity, tumor antigen deficiency, dysfunction of antigen-presenting cells, impaired T cell infiltration, activation of immunosuppressive signaling pathways, and enhanced immunosuppressive metabolism [24]. These mechanisms drive the transformation of the tumor-suppressive microenvironment into a tumor-promoting phenotype, which in turn induces tumor immune tolerance and facilitates tumor immune escape [25]. The altered expression of immune checkpoints and the activation of immunosuppressive signaling pathways both play crucial roles in the formation and function of the TME. For example, tumor cells recruit and secrete growth factors such as TGF-β, platelet-derived growth factor (PDGF), and IL-6, which stimulate fibroblasts to transform into CAFs. CAFs indirectly alter the body’s anti-tumor immunity by upregulating the expression of immune checkpoints such as PD-1/PD-L1, inducing T cell dysfunction and immune tolerance [26]. Immunotherapy based on ICIs is an emerging cancer treatment strategy that interrupts this immunosuppressive pathway and prevents or even reverses the establishment of immune tolerance, thus demonstrating long-lasting and potent anti-tumor activity in specific patients with different types of tumors [27].

**Figure 1.**
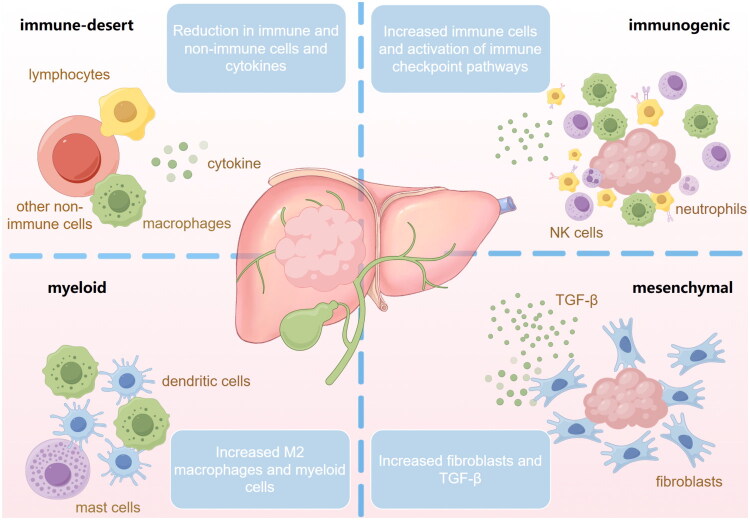
Immunological classification of intrahepatic cholangiocarcinoma. Based on the characteristics of the tumor microenvironment, ICC can be classified into four subtypes: immune-desert subtype, immunogenic subtype, myeloid subtype, and mesenchymal subtype. The immune-desert subtype is characterized by minimal infiltration of immune and non-immune cells and extremely weak expression of cellular molecules. The immunogenic subtype exhibits a microenvironment rich in innate and adaptive immune cells, with strongly activated immune checkpoint pathways. The myeloid subtype is characterized by high expression of monocyte-derived cells and fewer lymphocytes. In the mesenchymal subtype, activated fibroblasts exhibit strong expression, characterized by robust TGF-β expression and extracellular matrix remodeling.

ICC is characterized by a prominent desmoplastic TME, exhibiting significant immune suppression. Similar to other tumors, its TME also contains various non-immune and immune cell types, such as CAFs, TAMs, and abundant extracellular matrix components. These components promote tumor progression and metastasis through different mechanisms, such as immune suppression, induction of angiogenesis, and lymphangiogenesis [28]. ICC is markedly heterogeneous, and exhibits unique phenotypic differences in immune cell infiltration, genetics and extracellular matrix [29]. Job et al. classified ICC into four immunological subtypes (I1–I4) based on gene expression characteristics and immunohistochemistry (Figure 1) [30]. The I1 subtype exhibits an immune desert pattern, characterized by very weak infiltration and expression of cellular molecules in all immune and non-immune cells, with only a small number of stromal cells present in the surrounding area. The I2 subtype exhibits a reactive immunogenic pattern. A large number of innate and adaptive immune cells are recruited, with strong activation of inflammatory and immune checkpoint pathways. The I3 subtype, abbreviated as the myeloid-rich subtype, is characterized by moderate to high expression of monocyte-derived cells, particularly M2-type macrophages and myeloid cells, while lymphocytes and fibroblasts exhibit lower expression. The I4 subtype exhibits mesenchymal features, with strongly activated fibroblasts, exhibiting strong TGF-β expression and extracellular matrix remodeling. The immune landscape and immune evasion mechanisms vary significantly among different ICC subtypes based on the TME, indicating that each ICC subtype requires specific treatment strategies. This study found that the I1 subtype, which exhibits characteristics consistent with ‘cold tumors’, is the most prevalent in ICC. Similarly, another clinical study based on extensive transcriptomic data also validates this characteristic [29]. Current therapeutic strategies targeting ‘cold tumors’ may be more suitable for I1subtype, including the use of various modulators to convert them into inflammatory tumors that are sensitive to cancer immunotherapy [31]. The expression of multiple immune checkpoints in I2 subtype causes T cell exhaustion, preventing infiltrating T cells from mounting an effective immune response, ultimately leading to tumor progression. In contrast, its responsiveness to checkpoint blockade immunotherapy is significantly higher than that of other subtypes. M2 macrophages can suppress anti-tumor immunity, stimulate angiogenesis, and enhance tumor cell invasion, motility, and endophytic growth, enabling immune evasion and distant metastasis in I3 subtype. For the TME of this subtype, drugs like bisphosphonates and colony-stimulating factor 1 receptor inhibitors could be induce depletion or reprogramming of M2 macrophages, ultimately enhancing the antitumor immune response of host. I4 subtype has the poorest prognosis, possibly due to high levels of vascular factors (e.g. VEGF, FGF), chemokines (e.g. IL-6, TGF-β), and other factors produced by activated fibroblasts (e.g. αSMA), which may enhance pathways mediating primary tumorigenesis and inhibit immune cell recruitment into the tumor tissue. Moreover, the abundant fibrous matrix acts as a barrier, which may be related to the reported poor response to immunotherapy in this type, and this condition may be improved by antifibrotic therapy [30].

**Figure 2.**
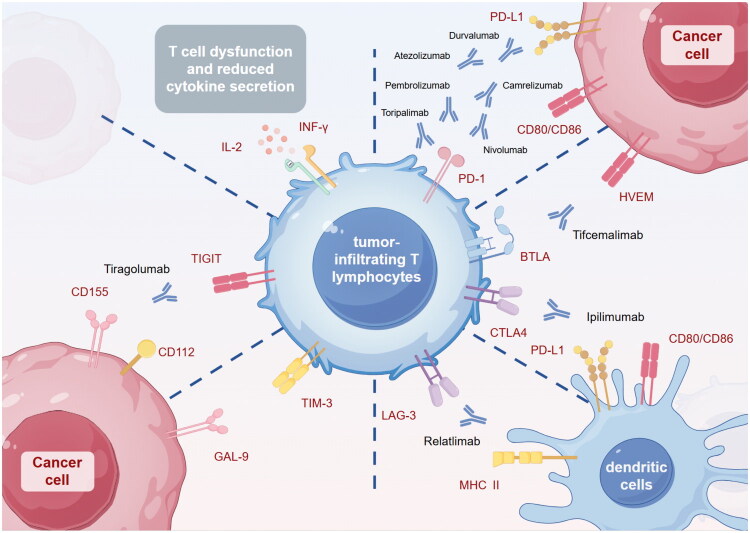
Inhibitory immune checkpoints in the immune microenvironment of intrahepatic cholangiocarcinoma. Under tumor conditions, inhibitory immune checkpoints on cancer cells exert their inhibitory effects on T cells through ligand-receptor binding. The primary inhibitory immune checkpoints include PD-1/PD-L1, CTLA-4, BTLA/HVEM, LAG-3, TIM-3, and TIGIT. Inhibitors targeting these immune checkpoints can disrupt this immune tolerance mechanism, blocking or even reversing the process, thereby demonstrating durable and potent antitumor activity across various tumor types. Among these, PD-1 can bind to PD-L1 and CD80/CD86 on cancer cells and APCs; CTLA-4 can competitively bind to B7 on APCs and also bind to CD80/CD86; BTLA binds to HVEM; LAG-3 binds to MHC II on APCs; TIM-3 binds to GAL-9 and others; TIGIT binds to CD155 and CD112. Table 1 :Preclinical and clinical studies of the expression of immune checkpoints in ICC.

To date, a multitude of studies have identified the expression of immune checkpoints in ICC (Figure 2), and research on the expression and mechanism of action of immune checkpoints is gradually increasing (Table 1). Nakamura et al. performed whole-exome sequencing on 260 patients with BTC and found that most patients overexpress inhibitory immune checkpoints, including PD-1/PD-L1, CTLA-4, and lymphocyte activation gene-3 (LAG-3) [42]. Subsequently, emerging immune checkpoints such as T cell immunoglobulin domain and mucin domain-3 (TIM-3) and T cell immune receptor with Ig and ITIM domains (TIGIT) have also been shown to be highly expressed in ICC and closely associated with the exhaustion of lymphotoxic immune cells (CD8 + T cells and NK cells) in the TME [32]. This experiment not only demonstrated the presence of other immune checkpoints in ICC, but also validated the co-expression of multiple immune checkpoints, which suggested that combining different ICIs may yield superior outcomes compared to monotherapy, and thus providing a theoretical foundation for dual immunotherapy [34,43]. Additionally, in I3 subtype, proliferating Tregs and myeloid cells also express various immune checkpoints such as PD-1 and CTLA-4, which interact with receptors ex­pressed on tumor-infiltrating immune cells, further promoting the formation of an immunosuppressive environment [33,37,44].

**Table 1.** The expression of immune checkpoints in ICC.

Molecule	Cellular expression	Ligand	Cellular expression	Reference
PD-1	CD8 + T cell	PD-L1	Tregs CD8 + T cell	[32]
	Cytotoxic T lymphocyte	PD-L1	Tumor-associated macrophages	[33]
	Tumor infiltrating lymphocyte	PD-L1	Tumor cell	[34]
	Tumor infiltrating lymphocyte	PD-L1	Tumor cell	[32]
	CD8 + T cell	PD-L1	Tumor cell interstitial cell	[35]
	CD4+ and CD8+ T cell	PD-L1	Tumor cell	[36]
CTLA-4	Tumor infiltrating lymphocyte	CD80/CD86	Antigen-presenting cell	[32]
	Tregs	CD80/CD86	Antigen-presenting cell	[37]
HVEM	Tumor cell	BTLA	CD8 + T cell	[32]
	Mesenchymal stem cells	BTLA	CD8 + T cell	[38]
	Tumor cell	CD160	Tumor-infiltrating lymphocytes	[39]
LAG-3	T cell	MHC II	Antigen-presenting cell	[32]
TIM-3	Tumor infiltrating lymphocyte	Galectin-9	Tumor cell	[32]
	Tumor infiltrating lymphocyte	Galectin-9	Tumor cell	[40]
TIGIT	CD8+ T cell	CD155	Tumor cell	[40]
	Tumor infiltrating lymphocyte	CD155	Tumor cell	[41]

## Clinical application and limitations of ICIs

The emergence of ICIs has opened new avenues for the treatment of malignancies. Common ICIs targeted PD-1 (e.g. pembrolizumab, tislelizumab, nivolumab), PD-L1 (e.g. durvalumab), or CTLA-4 (e.g. ipilimumab) are already available on the market and have demonstrated durable antitumor activity across multiple tumors [45]. Numerous studies have begun evaluating the efficacy of ICIs in ICC antitumor therapy (Table 2). Most of these clinical trials are currently in Phase I/II, with a primary focus on advanced-stage patients, and their ultimate efficacy remains to be ascertained.

**Table 2.** Clinical studies on targeting immune checkpoints in cholangiocarcinoma.

Intervention	Target	Trial phase	Number of patients	ORR (%)	OS (months)	PFS (months)	Reference
**ICIs monotherapy**							
Nivolumab	PD-1	I	30	NE	5.2	1.4	[46]
Nivolumab	PD-1	II	54	22.0	14.2	3.68	[36]
Pembrolizumab	PD-1	Ib	23	13.0	5.7	1.8	[47]
Pembrolizumab	PD-1	II	104	5.8	9.1	2.0	[48]
Durvalumab	PD-L1	I	42	4.8	8.1	1.5	[49]
**ICIs combination therapy**							
Camrelizumab external radiotherapy	PD-1	II	36	61.1	22.0	12.0	[50]
Camrelizumab GEMOX	PD-1	II	30	40.0	17.0	7.5	[51]
Nivolumab GC	PD-1	II	75	59.4	10.6	6.6	[52]
Nivolumab GC	PD-1	I	30	36.7	15.4	4.2	[46]
Pembrolizumab GEMOX	PD-1	II	11	27.3	9.9	4.1	[53]
Pembrolizumab GC	PD-1	III	1564	27.7	12.7	6.5	[21]
Durvalumab GC	PD-L1	III	685	NE	12.8	7.2	[54]
Durvalumab Tremelimumab	PD-L1 CTLA-4	I	65	10.8	10.1	1.6	[49]
Ipilimumab Nivolumab	CTLA-4 PD-1	II	39	31.0	5.7	2.9	[55]
Tiragolumab Atezolizumab	TIGIT PD-L1	I	3	NE	NE	2.8	[56]
Pembrolizumab Lenvatinib	PD-1	II	31	10.0	8.6	6.1	[57]
Camrelizumab Apatinib	PD-1 VEGFR-2	II	21	19.0	13.1	4.4	[58]
Rilvegostomig GC	PD-1/TIGIT	II	30	31.0	NE	8.3	[59]
Toripalimab Lenvatinib GEMOX	PD-1	II	30	36.7	15.4	4.2	[60]
Sintilimab Anlotinib GC	PD-1	II	50	52.8	13.2	9.1	[61]
Tislelizumab Donafenib GEMOX	PD-1	II	13	25.0	NE	4.2	[62]

Note: ORR objective response rate, OS overall survival, PFS progression free survival, GEMOX Gemcitabine plus Oxaliplatin, GC Gemcitabine and Cisplatin.

### ICIs monotherapy

At present, immunotherapy has made significant progress in the treatment of malignant tumors. ICIs, represented by anti-PD-1/PD-L1 inhibitors, have shown promising results in various types of cancer, including melanoma [63], NSCLC [64], and HCC [65]. The Phase III RATIONALE-301 clinical trial, which enrolled 674 patients with HCC, evaluated the efficacy and safety of tislelizumab as first-line treatment for unresectable HCC, with results showing a mOS of 15.9 months, a mPFS of 2.1 months, and an objective response rate (ORR) of 14.3% [66]. Similarly, anti-PD-1/PD-L1 inhibitors have also shown potential effectiveness in ICC. The Phase Ib KEYNOTE-028 trial, which evaluated the efficacy of pembrolizumab in 23 advanced BTC patients, demonstrated that when this drug was used as second-line therapy, it achieved a mOS of 5.7 months and a mPFS of 1.8 months [47]. The subsequent Phase II non-randomized KEYNOTE-158 trial assessed outcomes in high microsatellite instability (MSI-H)/mismatch repair (MMR) BTC patients treated with pembrolizumab monotherapy, which demonstrated that the mOS and mPFS were 7.4 and 2.0 months, with an ORR of 5.8% (6/104, all partial responses [PR]) [67]. A Phase II single-arm trial evaluated the efficacy of Nivolumab, with a mOS of 14.24 months, a mPFS of 3.68 months and an ORR of 11.0% [36]. Whereas, since most of these studies are in the Phase I/II stage with a small number of enrolled patients, there may be biases leading to significant differences in results, necessitating further analysis through large-scale randomized studies. Moreover, compared to other malignant tumors, such as melanoma [68] and Hodgkin’s lymphoma [69], the efficacy of anti-PD-1/PD-L1 monotherapy in treatment of advanced ICC remains limited [70].

Compared to anti-PD-1/PD-L1 inhibitors, monotherapy with anti-CTLA-4 inhibitors, such as ipilimumab, has only shown positive results in clinical trials for melanoma [71]. Since then, clinical trials of anti-CTLA-4 inhibitors have generally opted for combination therapy with anti-PD-1/PD-L1 inhibitors. Additionally, we do not find any clinical trials on single-agent (anti-CTLA-4 inhibitors) therapy for ICC. This indirectly suggests that monotherapy with anti-CTLA-4 inhibitors is ineffective in ICC. In the TME of ICC, CTLA-4 and PD-1/PD-L1 exhibit synergistic effects [43], which reveals that combination strategy of anti-CTLA-4 inhibitors and anti-PD-1/PD-L1 inhibitors may hold greater therapeutic potential [72].

Currently, no clinical trials targeting LAG-3 and TIM-3 have been reported in the field of ICC treatment. However, multiple preclinical study results indicate that LAG-3 and TIM-3 exhibit significantly high expression in ICC, and their expression levels are closely associated with patient clinical outcomes [40,73], which suggests that LAG-3 and TIM-3 may serve as novel potential targets for ICC immunotherapy, and the related ICIs and their therapeutic value warrant further in-depth investigation.

In summary, although ICIs have demonstrated certain antitumor activity in various advanced tumors, with several FDA-approved for advanced solid tumors characterized by MMR or MSI-H, the use of ICI monotherapy in ICC is still limited due to the tumor’s unique properties and its inherent resistance propensity [24]. From the clinical perspective, compared with HCC, gastric cancer, and colorectal cancer, ICC is more similar to pancreatic cancer in that it is highly malignant and progresses rapidly, resulting in significantly shorter PFS and OS for patients. This leaves insufficient time for immunotherapy to exert its optimal therapeutic effect and leads to limited efficacy in related immunotherapy clinical trials, even when ICIs demonstrate positive effects. The change in survival time is also insufficient to produce statistical significance. From the molecular mechanism perspective, this phenomenon occurs due to complex regulation among multiple immune checkpoints, as well as the fundamental absence of T cells in certain TME (such as the I1 subtype). The abundant matrix secreted by fibroblasts also contributes to this phenomenon, as it forms a physical barrier that impedes immune cell infiltration into tumor tissue. Consequently, even when ICIs reverse T-cell dysfunction, they remain unable to exert their intended effects [30].

### ICIs combination therapy

#### Chemotherapy-based

At present, chemotherapy remains the standard treatment for advanced or metastatic ICC. Given the limited efficacy of immunotherapy monotherapy in ICC, the combination of immunotherapy and chemotherapy may yield better clinical benefits. At the recent ASCO Gastrointestinal Cancers Symposium, the first Phase III trial—TOPAZ-1 (NCT03875235)—was presented, marking the formal entry of immunotherapy for advanced BTC into a new phase of clinical application [54]. Among 685 previously untreated patients, compared with GC regimen alone, treatment with durvalumab combined with GC regimen reduced the mortality by 20%, with higher OS and ORR (12.8 vs. 11.5 months and 26.7% vs. 18.7%). While the absolute differences across these survival endpoints are fairly small, the tail trends observed in the survival curves remain encouraging. Another phase III clinical trial (KEYNOTE-966) evaluated the efficacy of pembrolizumab combined with the GC regimen, yielding an ORR of 29% and a prolonged OS of 1.8 months (mOS: 12.7 vs. 10.9) [21]. Lei et al. compared multiple PD-1 inhibitors plus chemotherapy to chemotherapy alone and found similar results (mOS: 10.7 vs. 9.3 months; mPFS: 6.3 vs. 3.8 months) [74]. Based on these evidence-based findings, the role of immunotherapy combined with chemotherapy in ICC treatment has been validated and is poised to become the new standard for first-line therapy in advanced ICC. Yet further consideration is needed to determine whether population-specific or population related factors might influence treatment efficacy [5].

##### Dual immunotherapy

In recent years, a meta-analysis of phase III clinical trials evaluating novel combination therapy regimens for unresectable HCC has demonstrated that dual immunotherapy combining anti-PD-1/PD-L1 and anti-CTLA-4 inhibitors exhibits promising antitumor activity [75]. Given the limited efficacy of monotherapy immunotherapy in ICC, we have particularly focused on the potential value of dual immunotherapy regimens in ICC. In a Phase II trial (CA209-538), researchers evaluated the efficacy of the combination therapy of ipilimumab with nivolumab in ICC, with a mOS of 5.7 months, a mPFS of 2.9 months, and an ORR of 23% [55]. Additionally, patients showed better tolerability, with only 15% experiencing grade 3 or higher adverse events. Preclinical studies have also demonstrated the therapeutic efficacy of dual immunotherapy, as trials employing dual CTLA-4/PD-1 blockade showed an increase in the number of activated CD8^+^Cxcr3^+^IFNγ^+^ T cells [72]. Therefore, the concurrent blockade of these two key immune checkpoints is highly likely to emerge as a major breakthrough in overcoming ICC resistance and improving treatment efficacy. Several clinical trials combining PD-1 monoclonal antibodies with CTLA-4 monoclonal antibodies are currently underway, such as NCT02834013, NCT04969887, and NCT03101566. Recently, the preventive and therapeutic effects of CTLA-4-PD-L1 chimeric protein vaccines were validated in a rat model of ICC induced by thioacetamide [76]. In this trial, tumor burden decreased in rats, and PD-L1 expression on tumor cells was reduced, offering hope as a new second-line treatment option.

Surprisingly, in addition to CTLA-4, anti-TIGIT inhibitors can also synergistically inhibit tumor growth in immunocompetent mice. Dmitrij Ostroumov et al. found anti-TIGIT inhibitors can produce a synergistic inhibitory effect and inhibit the growth of liver cancer in mice with normal immune function, when combined with anti-PD-1 inhibitors [41]. In another phase I trial, A Japanese patient with advanced ICC received four cycles of combined therapy with atezolizumab (anti-PD-1 inhibitor) and tiragolumab (anti-TIGIT inhibitor), demonstrating better tolerability and antitumor activity (PFS: 2.8 months) [56]. However, the sample size of this trial is too small, and the presence of individual variability remains uncertain. More compelling evidence comes from a Phase II clinical trial (NCT05775159) evaluating rilvegostomig (PD-1/TIGIT bispecific antibody) combined with chemotherapy for advanced ICC, which showed a mPFS of 8.3 months and ORR of 31% in the rilvegostomig plus chemotherapy group, demonstrating good efficacy and acceptable safety [59].

In addition, targeting stimulatory immune checkpoints in the TME of ICC also demonstrates good response rates. The T cell exhaustion model constructed by L. P. Diggs et al. confirmed that anti-CD40/PD-1 significantly reduced tumor burden [77]. Experimental observations further revealed that CD40-mediated immune cell activation enhanced the anti-PD-1 response in ICC mice, indicating that targeting stimulatory immune checkpoints can also enhance the blocking effect of PD-1 inhibitors. Compared with the use of GC regimen alone, the combination of anti-CD40/PD-1 antibodies with GC regimen significantly improved survival rates in this trail [77].

##### Targeted therapy-based

Molecularly targeted therapies have also played a significant role in the treatment of advanced solid tumors, including receptor tyrosine kinase inhibitors (TKIs, such as lenvatinib, and sorafenib), VEGFR antagonists (such as bevacizumab) [78]. Sorafenib and lenvatinib were approved by the FDA for first-line treatment of HCC in 2007 and 2018, respectively [79,80]. Novel TKIs such as taletrectinib also have demonstrated encouraging efficacy and favorable safety profiles in ROS1^+^NSCLC patients, with an ORR of 88.8% and a mPFS of 45.6 months in treatment-naive patients [81]. Bevacizumab, as a key component of first-line therapy, has demonstrated significant clinical benefits in numerous malignancies (e.g. NSCLC, breast cancer), and a meta-analysis further revealed its superior efficacy in both mOS and mPFS compared to the control group [82]. Furthermore, an increasing number of clinical trials evaluating the efficacy of targeted therapies in ICC have emerged. In a Phase II study of lenvatinib as second-line therapy for unresectable BTC, the mOS was 7.35 months, the mPFS was 3.19 months and the ORR was 11.5% [83]. However, compared to the significant efficacy of lenvatinib in HCC (mOS: 13.6 months; mPFS: 7.6 months), its therapeutic benefit in BTC remains unsatisfactory [84].

Moreover, ICC exhibits unique mutation enrichments, such as IDH1/2 mutation, NTRK gene fusions, FGFR2 fusions/rearrangements, and RET fusions, particularly IDH1 mutations, which have higher mutation frequencies than other tumor types [85]. For patients who had IDH1-mutant ICC, ClarIDHy, a multicenter phase III study, validated the therapeutic efficacy of ivosidenib (IDH1 mutation inhibitor), reporting a mOS of 10.8 months and a mPFS of 2.7 months [86]. Among patients with BTC, FGFR2 fusions/rearrangements are found almost exclusively in ICC (accounting for 10%-15%) compared with extrahepatic cholangiocarcinoma [87]. Based on these characteristics, a pivotal Phase II clinical trial (FIGHT-202) enrolled 146 BTC patients who received daily pemigatinib showed that patients with FGFR2 fusions/rearrangements had improved mOS, mPFS and ORR (35.5%, 21.1, and 6.9 months, respectively) [88]. Based on this clinical trial, pemigatinib was approved by the FDA in 2020 for the treatment of advanced or metastatic ICC with FGFR2 fusions or rearrangements. At the subsequent 2021 ASCO-GI meeting, results from another Phase II clinical study of infigratinib (FGFR2 inhibitor) were reported, showing that in 108 patients with advanced BTC, the ORR reached 23.1% and the median duration of response (mDOR) was 5.0 months [89]. Although these inhibitors have provided clinical benefits to patients with specific mutations, response rates in some trails remain low. For example, in the ClarIDHy study, only 2% of patients treated with ivosidenib achieved partial response, with the disease control rate primarily driven by stable disease (51%) [86]. Preclinical evidence suggested that the IDH1 mutation may contribute to immunosuppressive signaling and that the depletion of CD8+ T cells diminishes the anti-tumor activity of IDH1 inhibitors [90], which raises the possibility of combining IDH1 inhibitors with immunotherapy. In a single-arm trial evaluating the combination of lapatinib and pembrolizumab for BTC (ICC of 50%), 12 patients (75%) diagnosed with ICC showed a positive treatment response, with a mOS of 11 months, a mPFS of up to 4.9 months, and an ORR of 25% [91]. Similarly, another Phase II trial evaluating anlotinib (multi-targeted TKI) combined with sintilimab (PD-1 inhibitor) as a first-line treatment regimen showed that, compared to conventional chemotherapy, the combination therapy yields an improved OS and PFS (OS: 15.8 months; PFS: 7.2 months) and safety was within an acceptable range [74].

##### Combined targeted therapy and chemotherapy

Given the complex mechanisms of resistance in ICC, researchers are exploring combination therapy regimens involving immunotherapy, targeted therapy, and chemotherapy, aiming to avoid resistance and enhance treatment efficacy. IMbrave151 (a Phase II, randomized, double-blind, placebo-controlled study) evaluated the efficacy of atezolizumab plus chemotherapy with or without bevacizumab in patients with advanced BTC (ICC of 54.9%), showing that the triple regimen has prolonged PFS (8.3 vs. 7.9 months), but did not yield a statistically significant improvement in OS (14.9 vs. 14.6 months) compared with the control group [92]. Another triple combination therapy of toripalimab, lenvatinib, and GEMOX also exhibited favorable efficacy in advanced ICC, with a mOS of 22.5 months and a mPFS of 10.2 months [60]. These findings collectively suggest that a triple regimen may emerge as a novel first-line treatment strategy for advanced ICC. Nevertheless, the combination of anti-PD-1 therapy with targeted therapy and chemotherapy led to a higher incidence of adverse events. Lei et al. demonstrated that the OS of the triple regimen (ICIs + chemotherapy + targeted therapy) is comparable to that of other dual regimens (14.4 vs. 10.7 and 15.8 months), whereas the incidence of grade 3 or higher adverse events was markedly increased (30.4% vs. 14.8% and 10.6%) [74]. Triple regimen for ICC requires further large-scale randomized controlled trials to validate its efficacy and safety in the future. Additionally, due to the high malignancy and rapid progression of ICC and the varying efficacy associated with different treatments, individualized treatment regimens are recommended to further optimize the prognosis of advanced ICC.

## Conclusion

To date, chemotherapy remains the primary treatment for advanced ICC. As research into immune mechanisms and immunotherapy in ICC continues to evolve, the role of immunotherapy is becoming increasingly evident. The TOPAZ-1 and KEYNOTE-966 trials have established immunotherapy plus chemotherapy as the new first-line standard treatment for advanced ICC. The relationship between targeted therapy and the immune system is also gradually being elucidated, offering potential as an adjunct to chemotherapy and immunotherapy. Nevertheless, individualized treatment selection remains essential. Further basic and clinical trials are needed to investigate the safety and efficiency of the ICIs and develop new combination therapy regimens.

## Date availability statement

Data sharing is not applicable to this article as no data were created or analysed in this study.
